# Sphingolipid Long-Chain Base Phosphate Degradation Can Be a Rate-Limiting Step in Long-Chain Base Homeostasis

**DOI:** 10.3389/fpls.2022.911073

**Published:** 2022-06-15

**Authors:** Benjamin Lambour, René Glenz, Carmen Forner, Markus Krischke, Martin J. Mueller, Agnes Fekete, Frank Waller

**Affiliations:** Pharmaceutical Biology, Julius-von-Sachs Institute of Biosciences, Julius-Maximilians-Universität Würzburg, Würzburg, Germany

**Keywords:** sphingolipid, long-chain base, plant sphingolipid metabolism, cell death, metabolic flux analysis, dihydrosphingosine-1-phosphate lyase, LC–MS/MS

## Abstract

Sphingolipid long-chain bases (LCBs) are building blocks for membrane-localized sphingolipids, and are involved in signal transduction pathways in plants. Elevated LCB levels are associated with the induction of programmed cell death and pathogen-derived toxin-induced cell death. Therefore, levels of free LCBs can determine survival of plant cells. To elucidate the contribution of metabolic pathways regulating high LCB levels, we applied the deuterium-labeled LCB D-erythro-sphinganine-d7 (D_7_-d18:0), the first LCB in sphingolipid biosynthesis, to Arabidopsis leaves and quantified labeled LCBs, LCB phosphates (LCB-Ps), and 14 abundant ceramide (Cer) species over time. We show that LCB D_7_-d18:0 is rapidly converted into the LCBs d18:0P, t18:0, and t18:0P. Deuterium-labeled ceramides were less abundant, but increased over time, with the highest levels detected for Cer(d18:0/16:0), Cer(d18:0/24:0), Cer(t18:0/16:0), and Cer(t18:0/22:0). A more than 50-fold increase of LCB-P levels after leaf incubation in LCB D_7_-d18:0 indicated that degradation of LCBs *via* LCB-Ps is important, and we hypothesized that LCB-P degradation could be a rate-limiting step to reduce high levels of LCBs. To functionally test this hypothesis, we constructed a transgenic line with dihydrosphingosine-1-phosphate lyase 1 (DPL1) under control of an inducible promotor. Higher expression of DPL1 significantly reduced elevated LCB-P and LCB levels induced by Fumonisin B_1_, and rendered plants more resistant against this fungal toxin. Taken together, we provide quantitative data on the contribution of major enzymatic pathways to reduce high LCB levels, which can trigger cell death. Specifically, we provide functional evidence that DPL1 can be a rate-limiting step in regulating high LCB levels.

## Introduction

Sphingolipid long-chain bases (LCBs) are the core building blocks of ceramides (Cers), which are processed into major plant membrane lipid families, glucosylceramides (GlcCers), and glycosylinositolphosphorylceramides (GIPCs). In addition, sphingolipids and LCBs are bioactive molecules involved in signal transduction processes in plants. Levels of specific LCBs have been functionally linked with processes such as stomatal closure (Ng and Hetherington, 2001; Coursol et al., 2003, 2005; Worrall et al., 2008; Dutilleul et al., 2012) and programmed cell death (Shi et al., 2007; Wang et al., 2008; Lachaud et al., 2010; Alden et al., 2011; Ternes et al., 2011; Magnin-Robert et al., 2015; Yanagawa et al., 2017; Glenz et al., 2019). High levels of LCBs are detected during programmed cell death reactions (Peer et al., 2010), and are also induced by the toxin Fumonisin B_1_, which serves as a virulence factor for necrotrophic pathogenic strains of the *Fusarium* clade (Abbas et al., 1994; Shi et al., 2007; Williams et al., 2007). As high levels of non-phosphorylated LCBs cause cell death (Shi et al., 2007; Glenz et al., 2019), regulation of free LCBs is crucial to prevent cell death in plants.

Levels of the two major LCBs in the model plant *Arabidopsis thaliana*, dihydrosphingosine (d18:0), and trihydrosphingosine (phytosphingosine, t18:0), are determined by the rate of LCB d18:0 synthesis from serine and palmitoyl-CoA [by Serine palmitoyltransferase 1 (SPT1) and 3-ketosphinganine reductase]. LCB d18:0 can then be hydroxylated to t18:0. LCBs are alkylated with fatty acids *via* an amide bond by ceramide synthases LAG1 HOMOLOG 1, 2, and 3 (LOH1, 2, and 3; Chen et al., 2009; Markham et al., 2011), resulting in ceramides. These ceramides are then further metabolized to the membrane lipids GlcCer or GIPCs. LCBs can also be phosphorylated by specific kinases (SPHK1/2 and LCBK1 and 2), which is required for their degradation by dihydrosphingosine-1-phosphate lyase (DPL1) to phospho-ethanolamine and hexadecanal (Tsegaye et al., 2007; Nishikawa et al., 2008). In addition to these reactions, LCB levels are influenced by the degradation of ceramides (ceramidase activity), or by dephosphorylation of LCB-Ps by phosphatases (SPP1; Imai and Nishiura, 2005; Worrall et al., 2008; Nakagawa et al., 2012).

When plants are exposed to Fumonisin B_1_, which blocks mainly the ceramide synthases LOH1 and LOH3, a 100-fold increase of LCB d18:0 is observed (Shi et al., 2007). Under these conditions of elevated LCB levels, also phosphorylated LCB species increase. This may indicate that DPL1, which requires phosphorylated LCBs for subsequent degradation, might be a bottleneck under these conditions. This is supported by the enhanced sensitivity of *dpl1* knockout plants against Fumonisin B_1_ (Tsegaye et al., 2007; Magnin-Robert et al., 2015; Glenz et al., 2019).

We were interested to identify the contribution of different enzymes for metabolizing LCBs, specifically when LCB levels are high, e.g., during plant cell death responses. Utilization of stable isotope labeled compounds has been proven a powerful tool to elucidate biosynthetic pathways and the metabolic fate of labeled compounds in the intact cellular environment (Hasunuma et al., 2010; Schwender et al., 2015; Allen, 2016). We therefore performed an experiment in which we fed stable isotope-labeled LCB d18:0 to *Arabidopsis* leaves and quantified labeled and non-labeled sphingolipids after 1, 3, 6, 24, and 48 h. We focused on immediate and abundant LCB-derived products that would have the most important impact on reducing high LCB levels. This data set enabled the quantification of isotopic incorporation rates and the assessment of the initial capacity of enzymes able to reduce high LCB d18:0 levels. To functionally address the contribution of the LCB-P degradation pathway, we constructed an inducible overexpressor line for DPL1 to test the significance of this specific LCB degradation pathway for regulating high LCB levels.

## Materials and Methods

### Plant Growth and Leaf Disk Feeding Conditions

*Arabidopsis thaliana* ecotype Colombia-0 (Col-0) as well as transgenic lines in the Col-0 background were sown in pots with soil and kept at 4°C for 2 days for stratification. Plants were then transferred to a growth chamber under a 9 h light/15 h dark cycle at 22/20°C (70% humidity) for 6 weeks. Twenty leaf disks per replicate were excised from 6-week-old *A. thaliana* Col-0 plants using a biopsy puncher (5 mm diameter) and were equilibrated in ultrapure water overnight. Leaf disks were then transferred to a control solution (H_2_O + 2% DMSO) or to a treatment solution [100 μM LCB D_7_-d18:0 (2S-amino-1,3R-octadecane-16,16,17,17,18,18,18-d7-diol; CAS Number: 1246304-35-7 obtained from Avanti Polar Lipids) + 2% DMSO]. Leaf disks were collected at 0, 1, 3, 6, 24, and 48 hpi, immediately frozen in liquid nitrogen and stored at −80°C for subsequent extraction of lipophilic compounds.

### Extraction of Lipophilic Compounds and Quantification of Sphingolipids

For the feeding experiment, lipid extraction was performed according to Shaner et al. (2009). In brief, frozen plant material was added to 2 ml screw cap tubes and ground with zirconia beads 3 times with a mixer mill and extracted using a solution containing 500 μl of butan-1-ol, 170 μl of H_2_O, 30 μl of citrate–phosphate-buffer pH 4.0, and 0.9 μl of four internal standards [d17:1, d20:1, d17:1-P, and Cer(d18:1/10:0)] at 0.1 μg/μl (18,000 g, 15 min; upper phase). The residual solution was extracted with a solution containing 165 μl butan-1-ol and 85 μl H_2_O (18,000 g, 15 min, upper phase). The combined upper phases were dried under vacuum (60°C, 2 h), and re-dissolved in 70 μl methanol:formic acid (99:1; v:v) for 30 min, centrifuged (18,000 *g*, 10 min), and then transferred to 300 μl glass vials, which were stored at −20°C until analysis by ultraperformance liquid chromatography (UPLC)-ESI-MS/MS. For the FB_1_ experiment, lipid extraction was performed as described in Glenz et al. (2019). Quantification of all samples was performed as described in Glenz et al. (2019). A detailed description is provided in Supplementary File 1.

### Construction of Inducible DPL1 Overexpression Lines

Coding sequence of *DPL1* (At1g27980) was amplified from cDNA, which was obtained by RNA extraction from Arabidopsis leaves with peqGOLD TriFast™ (VWR International LLC, Radnor, United States) and subsequent cDNA synthesis with M-MLV Reverse Transcriptase, RNase H Minus (Promega Corporation, Madison, United States), and Oligo(dT)27 primer, according to manufacturer instructions. Gateway® (Thermo Fisher Scientifc Inc., Waltham, United States) *attB* recombination sites were attached to the sequence with overlapping primers with *DPL1*-specific sequences ATGGATTCTTTTTCATATTCTTC and TTAATATTGACTGTCCATGAAAC by PCR amplification, using Phusion™ HF DNA polymerase (Thermo Fisher Scientifc Inc.). The amplicon was initially inserted in pGEM®-T Easy vector (Promega Corporation) and then transferred to pDONR™201 and destination vector pMDC7 (Curtis and Grossniklaus, 2003) using Gateway® technology (Thermo Fisher Scientifc Inc.), according to the manufacturer’s protocol. The vector pMDC7 was modified to contain a sequence for triple HA tag (Weiste and Dröge-Laser, 2014), resulting in an N-terminal fusion to the target sequence of *DPL1*. The resulting vector pMDC7ha-dpl1 was used for *Agrobacterium tumefaciens*-mediated transformation of *A. thaliana* ecotype Col-0 with floral dip (Clough and Bent, 1998). Selection of transgenic plants was done by growing seeds on medium supplemented with 40 μg/ml Hygromycin B (Duchefa Biochemie B.V., Haarlem, Netherlands). Transgenic plants were cultivated and propagated in soil and T_2_ generation was selected for single insertion of T-DNA by presumed 3:1 distribution of resistant/susceptible plants on Hygromycin B medium. Such selected lines were checked for homozygosity in T_3_ generation by Hygromycin B resistance and insertion of target sequence was confirmed by PCR. Expression of *DPL1* was determined by spraying leaves with β-estradiol (10 μM in water) and subsequent qRT-PCR. The selected transgenic line *XVE-HA-DPL1* allowed inducible expression of *DPL1*, as described by (Zuo et al., 2000).

### Fumonisin B_1_ Tests

For seedling tests, seeds were surface-sterilized with 70% ethanol, following sodium hypochloride (2.5% v/v) and water washing steps, and then sown on half-strength MS medium Murashige and Skoog (1962). The medium was supplemented with 0.5 μM Fumonisin B_1_ (Cayman Chemical, Ann Arbor, United States) and/or with 10 μM β-estradiol (Merck KGaA, Darmstadt, Germany) as indicated. Plates were incubated for 3 days at 4°C in the dark and then transferred to growth conditions as described for plants grown on soil. For leaf disk experiments, solutions were prepared by evaporating the appropriate amount of FB_1_ and/or β-estradiol stock solution, dissolving it in methanol and diluting it for the required concentration in H_2_O. Solutions did not exceed 2% (v/v) of methanol, and a 2% (v/v) methanol solution was used as control treatment. The solutions were used for floating leaf disks in cell death assays. In these assays, leaf disks (5 mm diameter) were detached from leaves of 6-week-old plants and floated on ultrapure H_2_O overnight. Subsequently, five leaf disks per sample were equilibrated in 0.3 ml of ultrapure H_2_O for 1 h prior to exchanging the water with the same volume of treatment solution. For sphingolipid quantification, leaf disks were collected at indicated time points, immediately frozen in liquid nitrogen, and stored at −80°C for subsequent extraction. Conductivity of the solution was determined using a LAQUAtwin EC-11 conductivity meter (Horiba, Kyoto, Japan). After the last measurement, the leaf disks were heated in the treatment solution in closed vials for 1 h at 100°C, and the conductivity representing 100% of cell death was determined after cooling the samples to room temperature.

## Results

### Dihydrosphingosine Is Mainly Metabolized to LCB t18:0, LCB-Ps, and Cer(d18:0/16:0)

**Figure 1:**
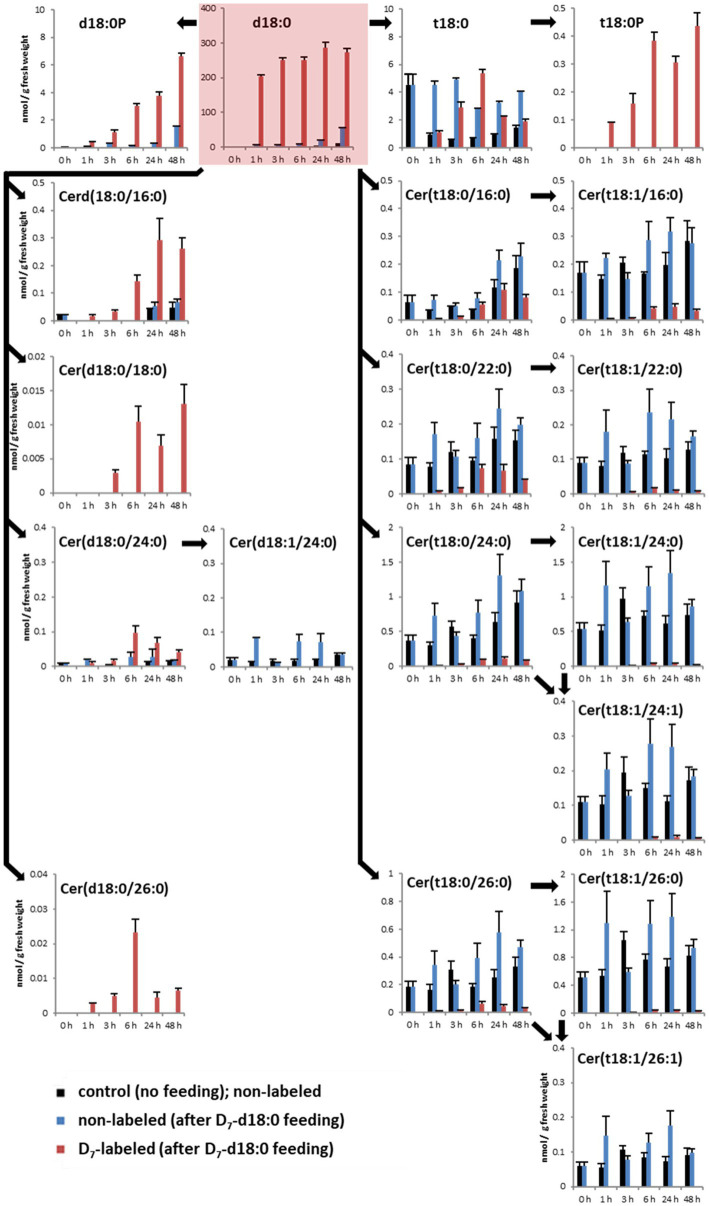
Quantification of major LCBs, LCB phosphates (LCB-Ps), and ceramides in the leaf after feeding LCB D_7_-d18:0. Arabidopsis leaf disks were incubated either in control solution (“control”, no feeding), or in 100 μM LCB D_7_-d18:0, and were harvested at indicated time points. 0 h refers to samples immediately before incubation, therefore samples for control and feeding are identical. D_7_-labeled and non-labeled metabolites were detected by ultraperformance liquid chromatography (UPLC)-MS/MS. Concentrations are given in nmol/g FW. Values are means of 4–6 biological replicates, with error bars indicating SE. Arrows indicate enzymatic reactions required for conversion of respective metabolites.

To identify the major metabolic routes which process free sphingolipid LCBs, Arabidopsis leaf disks were incubated in a solution containing deuterium-labeled LCB d18:0 (D_7_-d18:0), and were collected for quantification of major sphingolipids after 1, 3, 6, 24, and 48 h. For quantification, we used UPLC coupled to tandem mass spectrometry (MS/MS). We focused on those major sphingolipids that are formed from LCB D_7_-d18:0 by one, two, or three enzymatic reactions, which included major LCBs, LCB-Ps, and 15 ceramide species (Figure 1). The enzymatic reactions included sphingobase hydroxylation (formation of t18:0), LCB phosphorylation (formation of LCB-Ps d18:0P and t18:0P), ceramide synthesis (formation of ceramides with different fatty acid acyl chain lengths), and subsequent desaturation of LCB or fatty acid acyl chain of these ceramides.

To detect the contribution of the different enzymes processing high levels of LCB d18:0, we measured levels of D_7_-labeled sphingolipids after LCB application. The substrate, LCB D_7_-d18:0, was present in leaf disk samples at high levels of about 200 nmol/g fresh weight (FW) 1 h after incubation, and remained high (up to 274 nmol/g FW) until the end of the experiment after 48 h (Figure 1). One hour after incubation, the highest levels of isotope-labeled sphingolipids were the LCBs D_7_-t18:0 (1.1 nmol/g FW), D_7_-d18:0P (0.4 nmol/g FW), and D_7_-t18:0P (0.09 nmol/g FW), while the combined 15 D_7_-ceramide species accounted for 0.068 nmol/g FW, with D_7_-Cer(d18:0/16:0; 0.017 nmol/g FW) being the most abundant (Figure 1; Supplementary Table 1). Over the course of 48 h, steady-state levels of labeled Cers and LCB-Ps strongly increased, with D_7_-Cers reaching a combined total amount of about 0.65 nmol/g FW, while the two D_7_-LCB-P species reached 7 nmol/g FW (Figure 1).

### LCB-Ps and Different Groups of Ceramides Differ in Isotope Incorporation Rates

**Figure 2:**
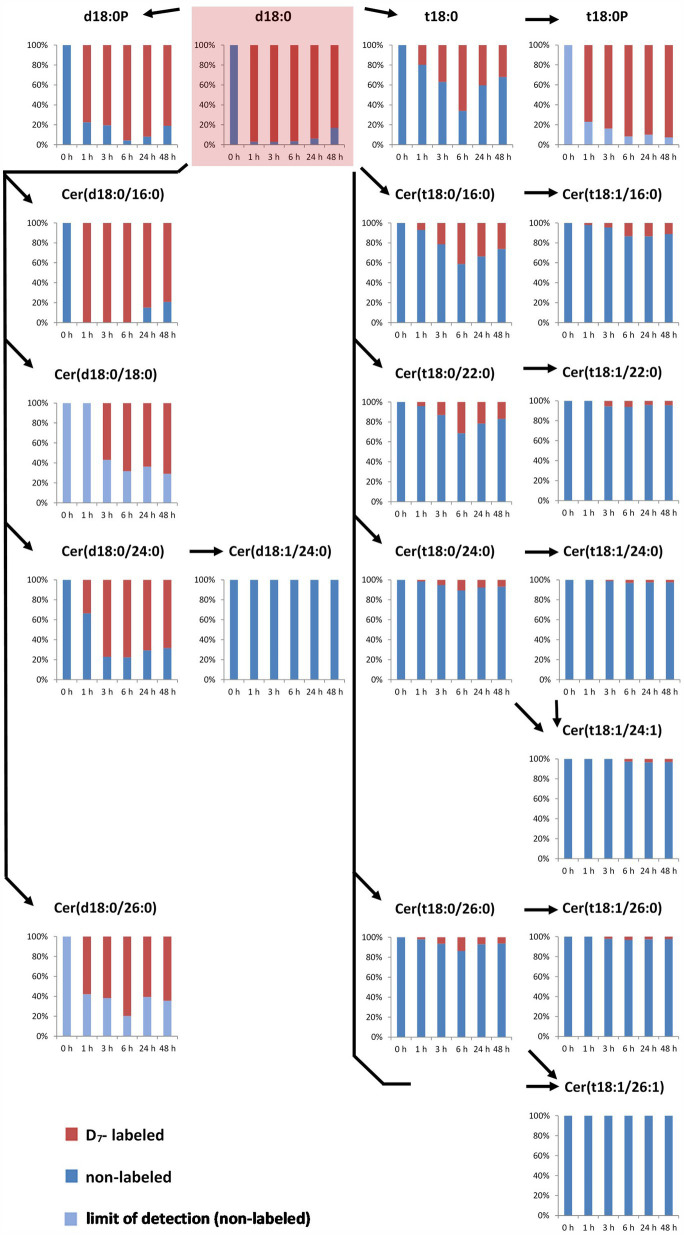
Accumulation of major labeled LCBs, LCB-Ps, and ceramides in the leaf after feeding LCB D_7_-d18:0. Arabidopsis leaf disks were incubated in 100 μM LCB D_7_-d18:0 and harvested at indicated time points. D_7_-labeled and non-labeled metabolites were detected by UPLC-MS/MS and relative amounts (in %) were calculated from concentrations of respective non-labeled and D_7_-labeled metabolites (presented in Figure 1). In case that respective non-labeled metabolites could not be detected, values for the respective limit of detection (LOD) were used for estimating the degree of labeling (light blue bars).

In the LCB D_7_-d18:0 incubated leaf samples, we quantified labeled and the respective non-labeled naturally occurring compounds. This enabled the calculation of the degree of labeling (mol%) of each compound over time. Initial labeling ratios (1 h) indicate the metabolic rate of incorporation for a given compound. At 1 h, 97 mol% of the total amount of free LCB d18:0 was labeled (Figure 1). For d18:0P, a high degree of labeling (77 mol%) was reached at 1 h, with values at later time points between 80 and 95 mol% (Figure 2). Hydroxylation of LCB D_7_-d18:0 to D_7_-t18:0 led to a degree of labeling between 20 (1 h) and 65 mol% (6 h; Figure 2). Interestingly, LCB t18:0P was labeled to a higher degree, between 75 (1 h) and 95 mol% (48 h). Labeling of Cers was as high as 60–100 mol% after 1–3 h for the d18:0-containing Cers [Cer(d18:0/16:0), Cer(d18:0/18:0), Cer(d18:0/24:0), and Cer(d18:0/26:0)]. Cers containing t18:0 or t18:1 reached lower levels between 4 mol% [e.g., Cer(t18:1/24:0) or Cer(t18:0/24:1)] and 40 mol% in case of Cer(t18:0/16:0) 6 h after feeding. For three labeled compounds detected in considerable amounts, the respective non-labeled species [t18:0P, Cer(d18:0/18:0), and Cer(d18:0/26:0)] were below the limit of detection (LOD). For this reason, the (minimum) degree of labeling was calculated by using the LOD values for these metabolites (Figure 2; Supplementary Table 1).

### Kinetics of Sphingolipid Levels After LCB d18:0 Feeding

Within the same experimental setup, we also included a mock treatment (2% DMSO without LCB d18:0), which allowed us to compare sphingolipid steady-state levels of leaves not incubated with LCB d18:0. Mock-treated samples showed comparable levels over time, with some ceramide species showing slightly increased levels at the 48 h time point. LCBs d18:0P, t18:0P, Cer(d18:0/18:0), and Cer(d18:0/26:0) were below the LOD in mock-treated samples. The highest increase of absolute amounts (combined steady-state levels of labeled and non-labeled compounds) at any of the five time points after treatment with LCB d18:0 were detected for d18:0P (8.2 nmol/g FW), t18:0 (8.1 nmol/g FW), and t18:0P (0.44 nmol/g FW), while most ceramide species had values less than 0.5 nmol/g FW. Interestingly, LCB D_7_-d18:0 feeding also resulted in a significant increase of some non-labeled species at later time points, e.g., LCBs d18:0 and d18:0P.

### Fumonisin B_1_-Induced Cell Death Is Reduced by Overexpression of Dihydrosphingosine Phosphate Lyase 1

**Figure 3:**
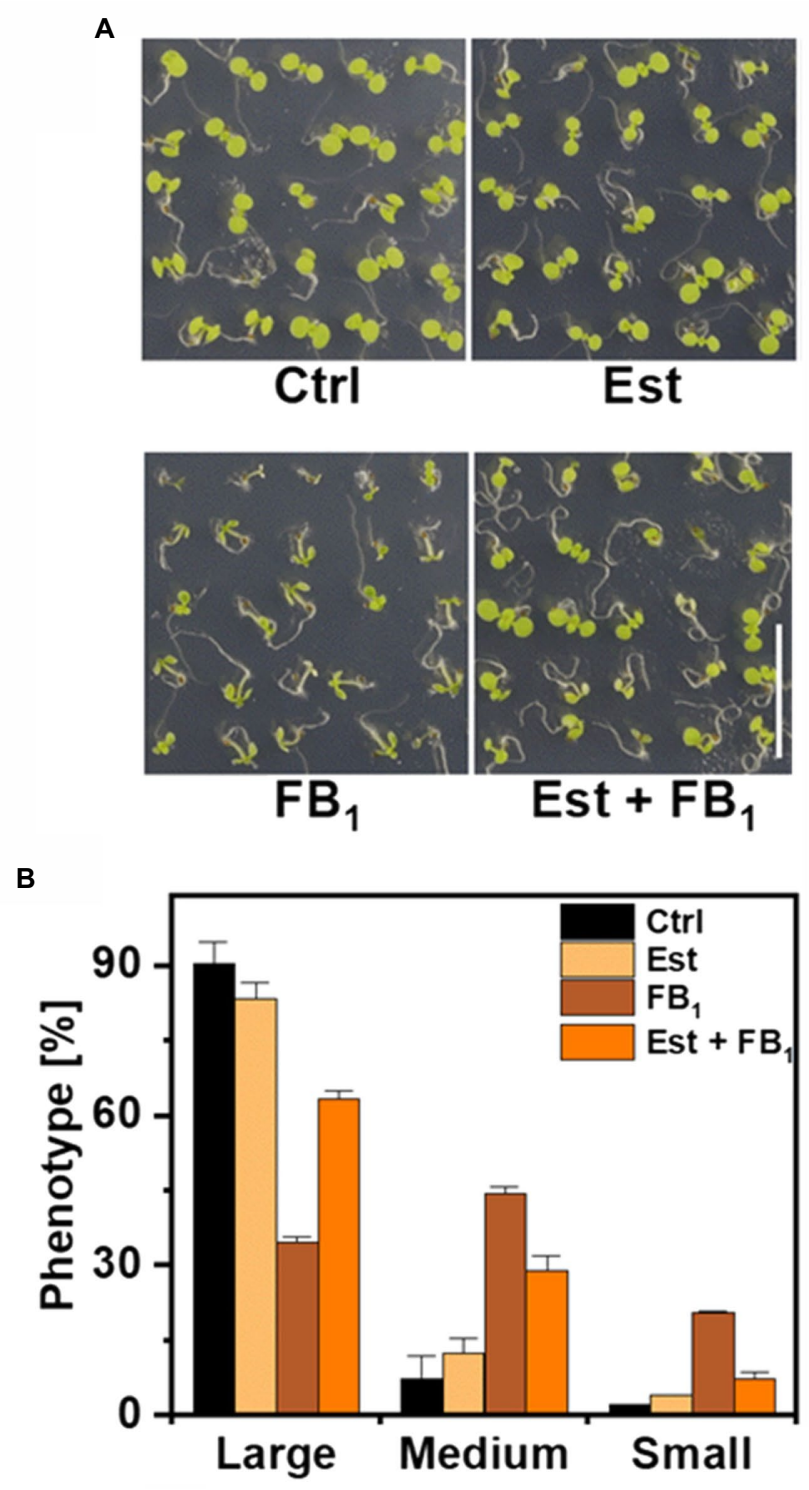
Effect of DPL1 overexpression on Fumonisin B_1_-induced growth inhibition in Arabidopsis seedlings. **(A)** Phenotypes of Arabidopsis line *XVE-HA-DPL1* after 12 days growth on control medium (Ctrl), medium containing 10 μM β-estradiol (Est), 0.5 μM FB_1_, or a combination of both (Est + FB_1_). Scale bar indicates 1 cm. **(B)** Categorization of the seedlings of *XVE-HA-DPL1*, treated as depicted in **(A)**, after 12 days of growth according to three groups: Large-, medium-, and small-sized plants. Relative frequencies of 49 seedlings per treatment were calculated. Values are means of two independent plates, with error bars indicating SD.

High levels and incorporation rates of LCB-Ps after feeding of LCB d18:0 prompted us to hypothesize that DPL1 (At1g27980) may play a crucial role in processing LCBs under conditions of high LCB levels. DPL1 converts LCB-Ps into hexadecanal and phosphoethanolamine, which are further metabolized. To elucidate the contribution of DPL1 to process LCB-Ps and thereby reduce high LCB levels, we constructed an Arabidopsis line (*XVE-HA-DPL1*) with the *DPL1* gene under control of a β-estradiol inducible promotor (Zuo et al., 2000). Using real-time PCR, we confirmed that β-estradiol was able to strongly increase *DPL1* transcript levels. To test the ability of this line to cope with high LCB levels, we grew seedlings on agar plates containing 0.5 μM FB_1_, which leads to strong reductions in growth due to accumulation of LCBs (Shi et al., 2007; Glenz et al., 2019). After 12 days on FB_1_ medium, plants were significantly smaller, as compared with plants on medium without FB_1_ (Figure 3A). Induction of DPL1 expression by addition of β-estradiol to the medium reduced growth inhibition by FB_1_, while estradiol alone had no effect, compared to control medium (Figure 3A). Growth effects were quantified by assigning plants to three size classes. FB_1_ treatment resulted in a shift to smaller plant size classes; while simultaneous estradiol treatment reduced the number of small plants (Figure 3B).

### Higher DPL1 Expression Reduces FB_1_-Induced High LCB Levels in the Leaf

**Figure 4:**
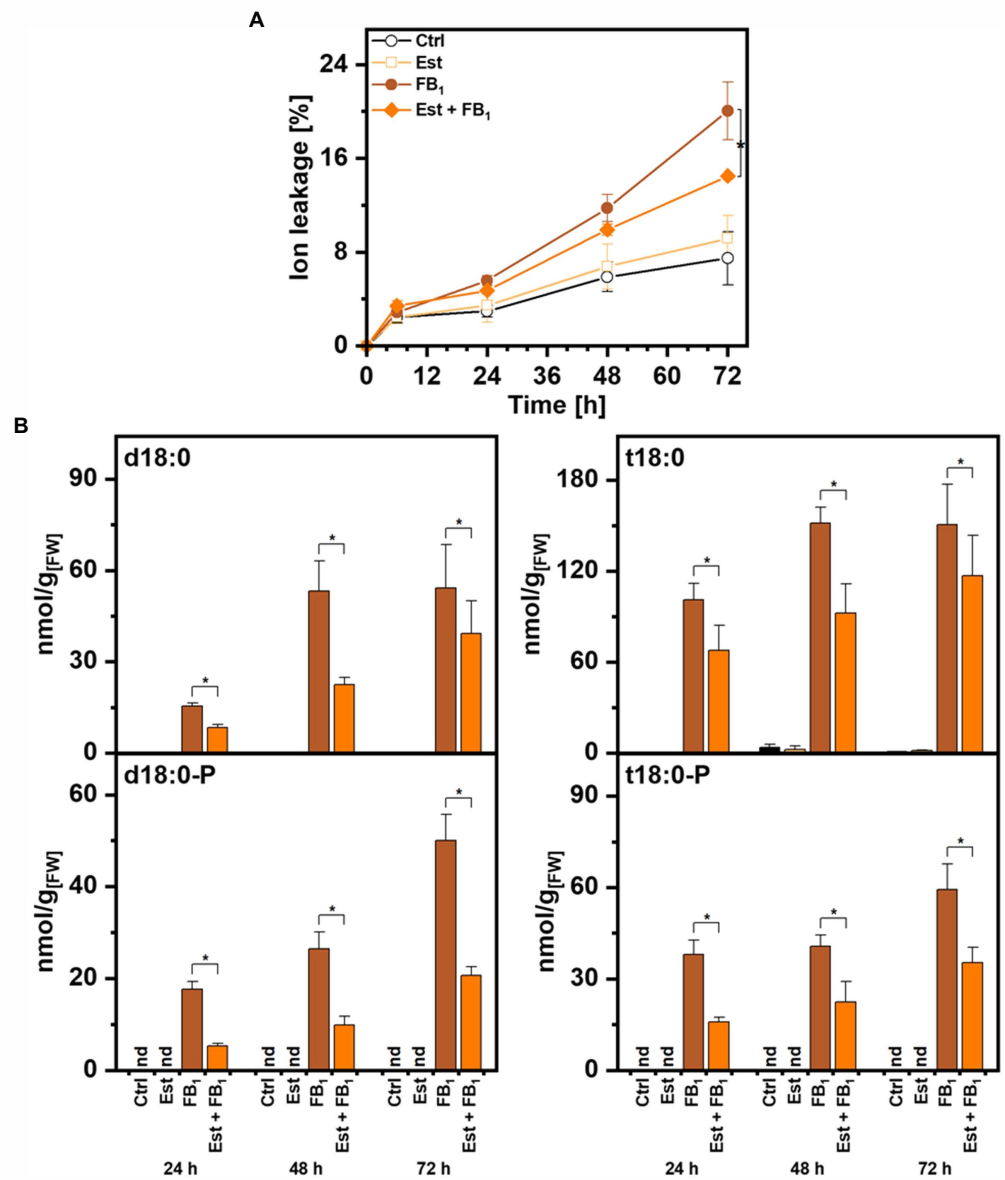
Effect of dihydrosphingosine-1-phosphate lyase 1 (DPL1) overexpression on Fumonisin B_1_-induced cell death and on LCB levels in Arabidopsis leaves. Leaf disks of Arabidopsis line *XVE-HA-DPL1* were incubated in control solution (2% methanol), 10 μM β-estradiol (Est), 100 μM Fumonisin B_1_ (FB_1_), or a combination of both (Est + FB_1_). **(A)** Time-course analysis of cell death, measured as the conductivity of the solution at respective time points relative to the conductivity of the boiled sample at the end of the experiment, which was set to 100%. **(B)** Levels of LCBs d18:0, d18:0P, t18:0, and t18:0P in leaf disks at indicated time points. Values are means of four independent samples, each consisting of five leaf disks. Error bars indicate SD. Asterisks indicate significance levels <0.05. For cell death measurements, these levels were calculated by ANOVA for the factor “Est treatment,” with Est and FB_1_ treatment as fixed factors and the factor “time” as a covariate. For LCB concentrations, significance levels were calculated for each of the four LCBs in a general linear model for the factor “Est treatment,” with the factor “time” as a covariate.

To quantify the contribution of higher expression of *DPL1* on LCB levels and on cell death induced by FB_1_, we performed tests with leaf disks of the Arabidopsis *XVE-HA-DPL1* line. To determine the induction of *DPL1* transcripts by β-estradiol under these conditions, we performed real-time quantitative PCR. Relative *DPL1* expression levels were 8–10 times higher in the samples treated with the inducer, irrespective of the presence of FB_1_. Leaf disk tests enable the quantification of cell death by measuring the conductivity of the solution. Compared to control treatments with 8–9% cell death, FB_1_ treatment induced cell death levels of 20% after 72 h, while simultaneous treatment with β-estradiol reduced these levels to 13% (Figure 4A).

To assess the enzymatic activity of DPL1, we quantified the levels of the LCBs d18:0 and t18:0 and LCB-Ps d18:0P and t18:0P by UPLC-MS/MS. As expected, FB_1_ treatment induced high levels of LCBs and LCB-Ps between 50 and 150 nmol/g FW after 72 h (Figure 4B). The combined treatment with FB_1_ and β-estradiol decreased levels of LCB-Ps, which are the substrates of DPL1, two- to three-fold at the three time points 24, 48, and 72 h (Figure 4B). LCB-P precursors LCB d18:0 and t18:0 also showed significantly reduced levels in the combined treatment. Enhanced *DPL1* expression therefore correlated with reduced LCB levels and reduced cell death induction.

## Discussion

### Feeding of Isotope-Labeled LCB D_7_-d18:0 to *Arabidopsis* Leaves and Subsequent Quantification by UPLC-MS/MS Enables Determination of LCB Product Turnover

High LCB levels in plants lead to cell death and are associated with programmed cell death reactions (Shi et al., 2007; Peer et al., 2010; Glenz et al., 2019). To regulate high LCB levels, plants have different options: downregulation of LCB *de novo* synthesis (as shown for ORM proteins; Gonzalez-Solis et al., 2020), LCB incorporation into sphingolipids by ceramide synthases (Markham et al., 2011), or LCB degradation by phosphorylation and subsequent degradation of LCB-Ps by DPL1 into hexadecanal and phosphoethanolamine (Tsegaye et al., 2007). To identify the contribution of those enzymes able to reduce high levels of LCBs, we applied saturating levels of isotope-labeled LCB D_7_-d18:0 to Arabidopsis leaves and subsequently quantified the major products derived from this compound. LCB D_7_-d18:0 levels in the treated leaves remained high throughout the time course experiment, mimicking high LCB levels induced by the fungal toxin Fumonisin B_1_ (Shi et al., 2007). The high degree of LCB d18:0 labeling in the leaf samples (97% after 1 h) enabled the quantification of LCB d18:0 products with high and low turnover. Measured initial incorporation rates (1 h; Figure 1), e.g., for Cer(d18:0/16:0) were in a similar range (0.02–0.04 nmol g^−1^ h^−1^) as reported in an experiment with ^15^N-labeled Serine for Arabidopsis seedlings (Shi et al., 2015). The high incorporation rates, e.g., into Cer(d18:0/16:0) or LCB d18:0P, indicated that the isotope-labeled LCB was readily taken up into the leaf cells and was available for enzymatic processing.

### LCB Phosphorylation Plays a Prominent Role in Initial Processing of High LCB Levels

**Figure 5:**
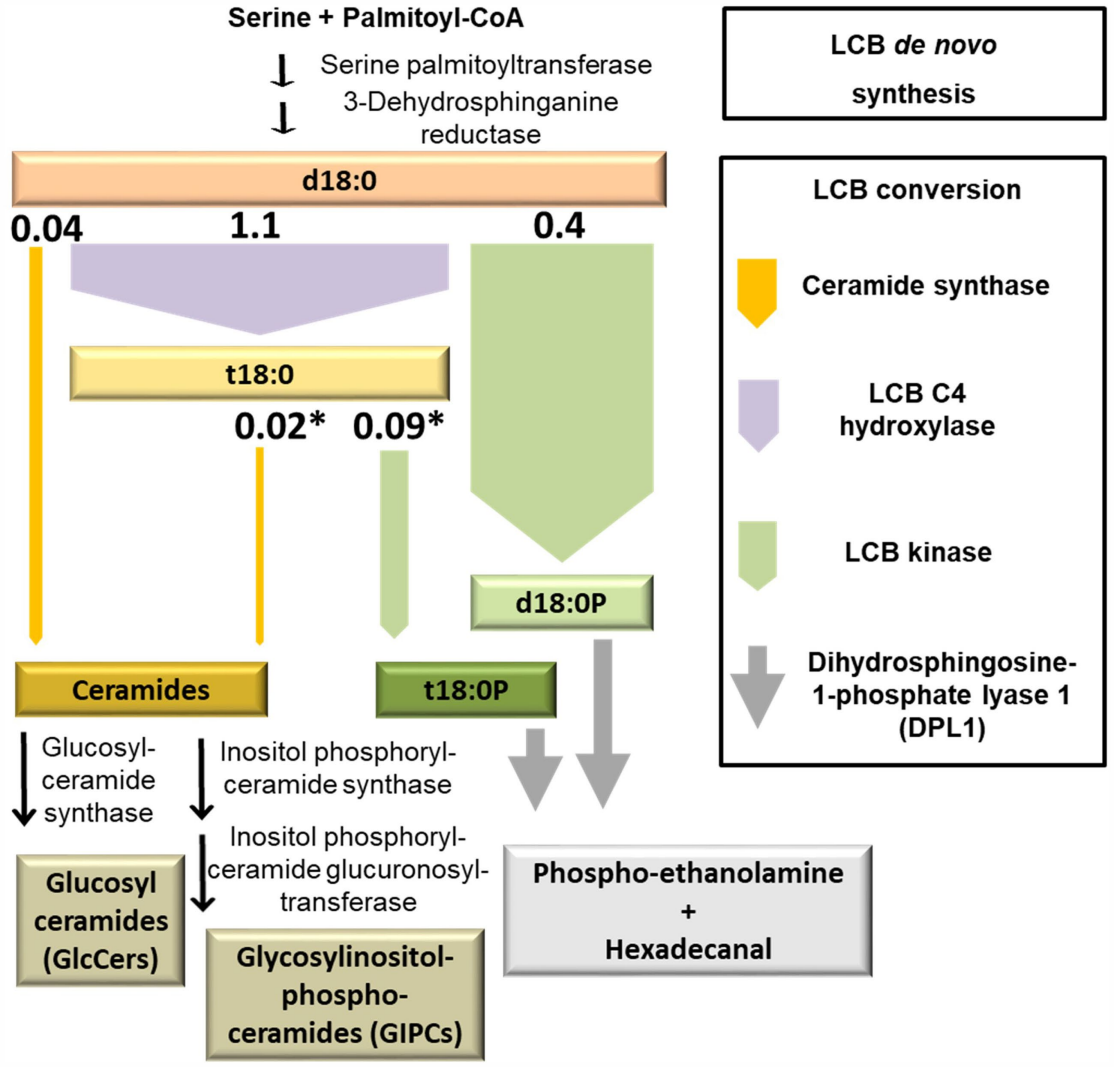
Model depicting the initial capacity of sphingolipid LCB-converting enzymes. Simplified diagram of plant sphingolipid metabolism, highlighting major LCB-converting enzymatic pathways. The width of the arrows represents the capacity of respective enzymes. This capacity was estimated from levels of D_7_-labeled LCB d18:0 products measured at the earliest time point, 1 h after incubation. The respective values (nmol/g FW; Figure 1) are depicted on top of the arrows. Asterisks indicate those reactions requiring a previous enzymatic step, which leads to an underestimation of the capacity due to the time required for processing the metabolic precursor.

Incorporation of the D_7_ label into compounds at an early time point after the onset of feeding can be used as a proxy for the initial capacity of those enzymes able to process LCB d18:0. The values are estimates, as further processing of labeled compounds is not accounted for. Values of this LCB processing capacity calculated from the detected amounts 1 h after the onset of LCB D_7_-d18:0 feeding are displayed in Figure 5. The highest capacity, with 1.1 nmol per g FW, was detected for sphingobase hydroxylase, which produces LCB t18:0. Phosphorylation of both labeled LCBs, d18:0 and t18:0 by LCB kinases resulted in d18:0P and t18:0P levels of 0.4 and 0.09 nmol/g FW at 1 h, respectively, indicating the significant contribution of this enzymatic step in d18:0 metabolism. In comparison, ceramide synthases contributed only about 0.068 nmol/g FW, as calculated from the amounts of 15 major ceramide species. Initial processing capacity for high LCB levels was therefore mainly provided by LCB kinases, while ceramide synthase capacity was more than 6 times lower (Figures 1, 5). Over the course of 48 h, levels of labeled LCB-Ps and of some Cers strongly increased, compared to the values at 1 h. Labeled ceramides reached a combined total amount of about 0.65 nmol/g FW, LCB-Ps had 10 times higher levels, with around 7 nmol/g FW after 48 h (Figure 1; Supplementary Table 1). Higher levels of these compounds at this late time point may indicate a higher activity of respective ceramide synthases and LCB kinases. However, both ceramides and LCB-Ps can be further metabolized: Cers are further processed into GlcCers and GIPCs, which are major membrane lipid components with much larger pool sizes compared to ceramides (Markham et al., 2006). LCB-Ps are degraded *via* DPL1 to hexadecanal and phosphoethanolamine. These two compounds were not recovered in our analysis. Therefore, we could not calculate flux rates of the D_7_ label into the different final degradation products in this experiment.

### LCB-Ps and Specific d18:0-Containing Cers Have the Highest Turnover Rates Among LCB d18:0-Derived Sphingolipids

The degree of labeling was highly different for the different sphingolipid species and followed a clear pattern over time. The high level of label detected in d18:0 containing Cers, e.g., Cer(d18:0/16:0) with more than 95 mol% labeling 1 h after feeding, could indicate a high turnover rate (Figure 2). With a concentration of 0.3 nmol/g FW Cer(d18:0/16:0) at 24 h, this compound is almost as abundant as all other measured Cers together at this time point. The two major LCB-Ps, d18:0P and t18:0P have about 10 times larger pool sizes (Figure 1), and reached very high labeling levels (about 90 mol% for d18:0P at 24 h). Interestingly, the t18:0P pool (0.45 nmol/g FW at 48 h) was labeled to more than 80 mol%, while the precursor LCB t18:0 never reached more than 65 mol% labeling. This could be explained if there is a spatial division between the pool of LCB D_7_-t18:0 available for *de novo* synthesis and a pool of non-labeled LCB t18:0 (which could be derived from the degradation of LCB t18:0 containing ceramides) not being available for phosphorylation. LCB t18:0-containing Cers reached a maximum of 40 mol% labeling Cer(t18:0/16:0) at 6 h, while those ceramides with longer fatty acid acyl chains displayed lower mol% labeling. Those Cers requiring subsequent LCB desaturation displayed significantly lower label incorporation, below 10 mol%, even at late time points. This subsequent decrease in label indicates the low turnover of these compounds and could also originate from a low activity of the respective enzymes. A low desaturase activity can be also observed for Cer(d18:0/24:0), which is labeled up to 75 mol%, while labeled Cer(d18:1/24:0) was below the LOD (Figure 2).

Our results for label incorporation during the first hour of inoculation (Figure 5) are in a similar range as those observed by Shi et al. (2015), who analyzed flux changes of N^15^-labeled Serine into LCBs and ceramides in Arabidopsis seedlings treated with salicylic acid. Flux distributions in Shi et al. (2015) show that LCBs, ceramides, and hydroxyceramides represented the highest fraction of total flux, while GlcCers had much larger pool sizes, but smaller metabolic fluxes with low incorporation rates (Shi et al., 2015). While the pool sizes of LCBs are comparatively small, very-long-chain ceramides and hydroxyceramides were deemed important due to their high flux contribution (Shi et al., 2015). Phosphorylated LCBs were not quantified, but the degradation *via* DPL1 had flux rates of about 11 nmol g^−1^(DW) h^−1^ in Shi et al. (2015). Rates of LCB D_7_-d18:0 incorporation into d18:0P and t18:0P measured here were 0.5 nmol g^−1^ (FW) h^−1^. Converting the levels to dry weight (assuming a factor of 10 resulting from about 90% water content), the rate measured in our assay would differ from that of Shi et al. (2015) only by a factor of two, which could result from the high d18:0 levels in our experiment, and differences in plant age and growth conditions.

### Levels of Labeled and Unlabeled Sphingolipids Respond to d18:0 Feeding

The artificial elevation of LCB d18:0 levels had different impacts on the levels of labeled and non-labeled LCBs, LCB-Ps, and ceramides. Total (labeled and non-labeled) LCB t18:0 levels of treated leaves were about 10 times higher at 6 h, compared to non-treated leaves (Figure 1), and about 3–4 times higher at 48 h. Initial levels of non-labeled LCB t18:0 at 0 h were 4–5 times higher than at later time points, which could result from a transient upregulation due to physical stress which had been observed in earlier experiments (Peer et al., 2010). Elevated levels of some non-labeled ceramides detected during the feeding experiment could result from a higher turnover of unlabeled sphingolipids with large pool sizes, e.g., degradation of GlcCer or GIPC species. A strong increase in total LCB-P levels was observed for d18:0P and t18:0P, with levels increasing more than 50-fold. This could be explained by enhanced activity of respective kinases, lack of respective phosphorylase activity, and/or by an insufficient ability of DPL1 to degrade high concentrations of LCB-Ps.

### DPL1 Can Be a Rate-Limiting Step in Reducing High LCB Levels

Previous studies with knockout mutants have shown that SPHK1 and DPL1 are required for reducing high LCB levels, e.g., after FB_1_ treatment (Tsegaye et al., 2007; Magnin-Robert et al., 2015; Qin et al., 2017; Yanagawa et al., 2017; Glenz et al., 2019). In addition, during biotic stress (mimicked by salicylic acid treatment), LCB degradation *via* DPL1 is the most variable, with simulated flux variability ranges more than 20 times higher than all combined ceramide synthesis reactions, which are required for further processing into GlcCers and GIPCs (Shi et al., 2015). The detected high capacity of LCB kinases to process free LCBs, and the high increase of LCB-P levels after feeding LCB d18:0 (Figure 1), prompted us to hypothesize that DPL1 could be a rate-limiting step for degradation of LCBs. To functionally test this hypothesis, we constructed a DPL1 overexpressing line and analyzed the ability of this line to cope with high LCB levels.

Both assays, a seedling growth inhibition assay and a leaf disk cell death assay, revealed that induction of higher DPL1 expression was able to protect plants from deleterious high LCB levels (Figures 3, 4). Higher DPL1 expression was correlated with lower LCB-P levels, and was also able to reduce LCB levels, which are responsible for inducing cell death in Arabidopsis leaves (Figure 4). These results support our hypothesis that DPL1 activity can be rate-limiting under conditions of high LCB levels. The hypothesis is in line with our previous work (Glenz et al., 2019): Higher expression of SPHK1 resulted in the expected higher levels of LCB-Ps, e.g., when leaves were treated with LCB t18:0, but the reduction of LCB levels upon FB_1_ treatment was weak, and did not result in protecting plants from detrimental effects (Glenz et al., 2019). Now, this can be interpreted as a result of insufficient activity of DPL1, at least under the growth conditions tested. DPL1 could therefore be an interesting candidate to elevate plant FB_1_ resistance, e.g., by expression under control of a pathogen-inducible promotor. Luttgeharm et al. (2015) have shown that constitutive overexpression of LOH2 or LOH3 can improve FB_1_ resistance by reducing LCB levels. However, LOH2 overexpression results in a severe dwarf phenotype, presumably due to accumulation of C_16_-FA ceramides, while LOH3 overexpression had less severe effects on the phenotype, with improved growth, but elevated salicylic acid level compared to wild-type plants (Luttgeharm et al., 2015).

## Conclusion

This study provides crucial information on the capacity of those enzymes immediately processing LCBs under conditions of high LCB levels. Besides the hydroxylation by SBH1 (producing LCB t18:0 from d18:0), these enzymes are LCB kinases (which produce LCB-Ps that are degraded by DPL1) and ceramide synthases (LOH1, 2, and 3), which produce ceramides that are further modified by desaturases and hydroxylases prior to incorporation into membrane-localized, highly abundant pools of GlcCers and GIPCs. High levels and high turnover rates of LCB-Ps detected here pointed to the relevance of LCB degradation *via* phosphorylation and subsequent degradation into hexadecanal and phosphoethanolamine by the enzyme DPL1. Our functional test with an inducible DPL1 line shows that indeed, DPL1 can be rate-limiting for the reduction of high levels of LCBs. The relevance of regulating high LCB levels is illustrated by the role of elevated LCB levels during programmed cell death reactions in Arabidopsis (Peer et al., 2010), and by the fungal toxin FB_1_ produced by strains of the plant pathogenic *Fusarium* clade, which induces host cell death by elevating levels of LCBs (Abbas et al., 1994). A better understanding and the controlled manipulation of DPL1 activity could also be a strategy to enhance plant resistance to specific Fusarium strains utilizing Fumonisin B_1_ as a virulence factor.

## Data Availability Statement

The original contributions presented in the study are included in the article/**Supplementary Material**; further inquiries can be directed to the corresponding author.

## Author Contributions

RG, MM, AF, and FW designed the experiments. BL, MK, and FW performed the feeding experiments. RG and CF performed the functional tests. BL, RG, MK, MM, AF, and FW analyzed the experimental data. All authors contributed to the article and approved the submitted version.

## Funding

This work was supported by a grant from the Deutsche Forschungsgemeinschaft (DFG) to FW (WA 2478/4-1). The metabolomics core unit was supported by the DFG (project numbers 179877739 and 316629583). The publication was funded by the German Research Foundation (DFG) and the University of Wuerzburg in the funding program Open Access Publishing.

## Conflict of Interest

The authors declare that the research was conducted in the absence of any commercial or financial relationships that could be construed as a potential conflict of interest.

## Publisher’s Note

All claims expressed in this article are solely those of the authors and do not necessarily represent those of their affiliated organizations, or those of the publisher, the editors and the reviewers. Any product that may be evaluated in this article, or claim that may be made by its manufacturer, is not guaranteed or endorsed by the publisher.

## Supplementary Material

Supplementary Table 1 Amounts of major LCBs, LCB-Ps and ceramides in the leaf after feeding LCB D_7_d18:0 (corresponding to Figures 1, 2).

Supplementary Table 2 List of detected sphingolipids, standards and compounds used.

Supplementary File 1 Detailed description of sphingolipid detection and quantification by LCMS, including supplementary references.
