# Supplementary material for: Sphingolipid Long-Chain Base Phosphate Degradation Can Be a Rate-Limiting Step in Long-Chain Base Homeostasis
Source: Front Plant Sci. 2022 Jun 15;13:911073. doi: 10.3389/fpls.2022.911073 (PMC9240600; doi:10.3389/fpls.2022.911073)
Supplement: Supplementary Table 1 — Amounts of major LCBs, LCB-Ps and ceramides in the leaf after feeding LCB D7d18:0 (corresponding to Figures 1, 2). [file Data_Sheet_1.pdf]

Supplementary file 1:
Detailed description of sphingolipid detection and quantification by LCMS; Supplementary references

For measurement and quantification of sphingobases and ceramides, four internal standards (IS; 30 ng of LCB d17:1, d20:1, d17:1P, and Cer(d18:1/10:0)) were added to each sample during SPL extraction. In addition, standards at 90 ng/ 70 µL (**Table: Standard mixture)** were directly transferred into glass vials as reference materials. Immediately before the measurement, all samples were transferred to an ultrasonic bath for 5 min. All samples and IS were measured by HPLC-MS/MS for quantification.

Table : Standard mixture

**Substances**	**Label**	**MW**	**CAS Number**
[C17]-D-*erythro*-Sphingosine	d17:1Δ4	285.3	6918-48-5
D-*erythro*-Sphingosine	d18:1 Δ4 (Sphingosine)	299.3	123-78-4
D-*erythro*-Sphinganine	d18:0 (Sphinganine)	301.3	764-22-7
D-*erythro*-Sphinganine-d7	D7-d18:0	308.3	1246304-35-7
D-ribo-4-hydroxysphinganine	t18:0 (Phytosphingosine)	317.2	388566-94-7
[C20]-D-*erythro*-Sphingosine	d20:1 Δ4	327.3	6918-49-6
[C17]-D-*erythro*-Sphingosine-1-Phosphate	d17:1Δ4-P	365.2	474923-27-8
D-*erythro*-Sphingosine-1-Phosphate	d18:1 Δ4-P	379.3	26993-30-6
D-*erythro*-Sphinganine-1-Phosphate	d18:0-P	381.3	19794-97-9
D-ribo-4-hydroxysphinganine-1-Phosphate	t18:0-P	397.3	383908-62-1
N-decanoyl-D-*erythro*-sphingosine	d18:1 Δ4-c10:0	453.4	111122-57-7
N-palmitoyl-D-erythro-Sphinganine	d18:0-c16:0	539.5	5966-29-0
N-palmitoyl-Phytophingosine	t18:0-c16:0	555.5	111149-09-8
N-oleoyl-D-erythro-Sphinganine	d18:0-c18:1(9Z)	565.5	34227-83-3
N-stearoyl-D-erythro-Sphinganine	d18:0-c18:0	567.6	2304-80-5
N-lignoceroyl-D-erythro-Sphingosine	d18:1 Δ4-c24:0	649.6	102917-80-6
N-nervonoyl- D-erythro-Sphinganine	d18:0-c24:1(15Z)	649.6	352518-80-0
N-lignoceroyl-Phytosphingosine	t18:0-c24:0	667.6	34437-74-6

UPLC-MS/MS

Chromatographic separation of the analytes was carried out by ultra-performance liquid chromatography System (UPLC®, Waters Corporation, Milford, MA, USA). Analytes were then ionized by electrospray ionization (positive ESI mode) and detected by tandem mass spectrometry using a Quattro Premier Triple Quadrupole mass spectrometer (Waters Corporation, Milford, MA, USA).

Chromatographic separation

Reversed phase chromatography was performed with an ACQUITY UPLC® BEH C18 column (2.1 x 50 mm; particle size 1.7 µm) with a VanGuard pre-column (BEH C18; 2.1 x 5 mm; particle size 1.7 µm; In-Line particle filter 0.2 µm; flow rate: 350 µL/min; Water Corporation). Eluent A (58 % Methanol v/v; 41 % H_2O v/v; 1 % Formic acid v/v; 5 mM Ammonium formate) and Eluent B (99 % Methanol v/v; 1 % Formic acid v/v; 5 mM Ammonium formate) were used for gradient elution of Sphingolipids are displayed in **Table HPLC gradient**. Autosampler UPLC® system temperature was 20 °C, the column temperature was 30 °C, and the volume of samples injected was 8 µL.

Table HPLC gradient

**Time (min)**	**Eluent A (%)**	**Eluent B (%)**
0.0	60	40
2.0	60	40
4.0	20	80
5.5	15	85
8.0	5	95
14.0	0	100
20.0	0	100
20.1	60	40
24.0	60	40

MS/MS conditions

After chromatographic separation, the compounds were analyzed in positive ESI mode and detected by multiple reaction monitoring (MRM). The device settings were as follows:

Ionization mode	+ES
Capillary voltage (kV)	3
Source temperature (°C)	120
Desolvation temperature (°C)	450
Cone gas flow - N_2 (L/h)	50
Desolvation gas flow - N_2 (L/h)	800

Mass to charge ratios (m/z) in MRM mode of the individual sphingolipids with a dwell time of 25 ms for each transition and the specific cone voltage and collision energy are shown below. Argon was used as collision gas for the collision induced dissociation (CID) with a flow rate of 0.3 mL/min. Mass to charge ratios of the precursors (parent ions) and products (daughter ions) of the analyzed sphingolipids are specified in MRM transition. MS/MS parameters of Deuterium (D_7)-labeled sphingolipids measured are listed below.

Table: MS/MS parameters of sphingolipids

Sphingolipids	MRM transition (m/z)	Cone voltage (V)	Collision energy (eV)
d17:1 (IS)	286.2 → 268.1	20	11
d18:1	300.2 → 282.2	22	17
d18:0	302.2 → 284.2	28	18
t18:1	316.1 → 298.2	22	20
t18:0	318.1 → 282.2	22	20
d20:1 (IS)	328.1 → 310.2	18	22
d17:1-P (IS)	366.1 → 250.1	22	15
d18:1-P	380.0 → 264.2	20	17
d18:0-P	382.1 → 284.1	30	15
t18:1-P	396.2 → 298.2	24	20
t18:0-P	398.1 → 300.0	40	15
d18:1-10:0 (IS)	454.4 → 264.2	11	22
d18:0-16:1	538.7 → 266.3	45	35
d18:0-16:0	540.5 → 266.3	36	37
t18:1-16:0	554.5 → 262.3	30	30
t18:0-16:0	556.5 → 264.3	30	30
d18:1-18:1	564.7 → 264.3	35	35
d18:0-18:1	566.7 → 266.3	36	37
d18:0-18:0	568.5 → 266.3	36	37
d18:1-20:0	594.6 → 264.2	30	30
t18:1-22:0	638.6 → 262.3	35	40
t18:0-22:0	640.6 → 264.3	35	40
d18:1-24:0	650.6 → 264.2	28	32
d18:0-24:1	650.7 → 266.3	45	35
d18:0-24:0	652.7 → 266.3	45	35
t18:1-24:1	664.6 → 262.3	35	40
t18:1-24:0	666.6 → 262.3	35	40
t18:0-24:1	666.6 → 264.3	35	40
t18:0-24:0	668.6 → 264.3	35	40
d18:1-26:0	678.7 → 264.2	30	32
d18:0-26:0	680.7 → 266.3	43	35
t18:1-26:1	692.7 → 262.3	35	40
t18:1-26:0	694.7 → 262.3	35	40
t18:0-26:0	696.7 → 264.3	35	40

Table: MS/MS parameters of labeled D_7 sphingolipids

Sphingolipids	MRM transition (m/z)	Cone voltage (V)	Collision energy (eV)
d17:1 (IS)	286.2 → 268.1	20	11
D7-d18:1	307.2 → 282.2	22	17
D7-d18:0	309.2 → 291.2	28	18
D7-t18:1	323.1 → 305.2	22	20
D7-t18:0	325.1 → 289.2	22	20
d20:1 (IS)	328.1 → 310.2	18	22
d17:1-P (IS)	366.1 → 250.1	22	15
D7-d18:0-P	389.1 → 291.1	30	15
D7-t18:0-P	405.1 → 307.0	40	15
d18:1-10:0 (IS)	454.4 → 264.2	11	22
D7-d18:0-16:1	545.6 → 273.3	45	35
D7-d18:0-16:0	547.5 → 273.3	36	37
D7-t18:1-16:0	561.5 → 269.3	30	30
D7-t18:0-16:0	563.5 → 271.3	30	30

D7-d18:1-18:1	571.5 → 269.2	30	30
D7-d18:0-18:0	575.5 → 273.3	36	37
D7-t18:1-22:0	645.6 → 269.3	35	40
D7-t18:0-22:0	647.6 → 271.3	35	40
D7-d18:0-24:0	659.7 → 273.3	45	36
D7-t18:1-24:1	671.6 → 269.3	35	40
D7-t18:1-24:0	673.6 → 269.3	35	40
D7-d18:0-h24:1	673.6 → 273.3	30	30
D7-t18:0-24:0	675.6 → 271.3	35	40
D7-d18:0-26:0	687.7 → 273.3	43	35
D7-t18:1-26:0	701.7 → 269.3	35	40
D7-t18:0-26:0	703.7 → 271.3	35	40
D7-d18:0-h26:0	703.7 → 273.3	30	30

Data processing was carried out with MassLynx V4.1 (Waters Corporation). The concentration of the analytes was based on the amount of material used and internal standard, using reference factors (RFs) based on authentic reference materials available.
A correction factor was calculated during the measurement of each experiment by a standard mix of sphingobases and ceramides (each of them concentrated at 300 ng/µL) and used for the evaluation of the respective experiment. Since most of the ceramides measured in this work were not commercially available in contrast to sphingobases measured, a RF adapted from the structurally closest sphingolipid available for the quantification was used.

Supplementary references

Fahy E., Sud M., Cotter D. & Subramaniam S. (2007) LIPID MAPS® online tools for lipid research. Nucleic Acids Research 35: W606-12.

Ivashikina N., Deeken R., Ache P., Kranz E., Pommerrenig B., Sauer N., Hedrich R. (2003) Isolation of AtSUC2 promoter-GFP-marked companion cells for patch-clamp studies and expression profiling. Plant J., 36:931-945.

Livak K.J., Schmittgen T.D. (2001) Analysis of relative gene expression data using real-time quantitative PCR and the 2(-Delta Delta C(T)) Method. Methods 25: 402-408.

Pedrotti L., Mueller M.J., Waller F. (2013) Piriformospora indica Root Colonization Triggers Local and Systemic Root Responses and Inhibits Secondary Colonization of Distal Roots. PLoS ONE 8(7): e69352. doi:10.1371/journal.pone.0069352

Sud M., Fahy E., Cotter D., Brown A., Dennis E.A., Glass C.K., Merrill A.H. Jr, Murphy R.C., Raetz C.R., Russell D.W., Subramaniam S. (2007) LMSD: LIPID MAPS® structure database. Nucleic Acids Research 35: D527-32.
