# Supplementary material for: Sphingolipid Long-Chain Base Phosphate Degradation Can Be a Rate-Limiting Step in Long-Chain Base Homeostasis
Source: Front Plant Sci. 2022 Jun 15;13:911073. doi: 10.3389/fpls.2022.911073 (PMC9240600; doi:10.3389/fpls.2022.911073)
Supplement: Supplementary Table 2 — List of detected sphingolipids, standards and compounds used. [file Table_1.pdf]

	sphingobases				ceramides														
	d18:0	t18:0	d18:0P	t18:0P	d18:0/16:0	t18:1/16:0	t18:0/16:0	d18:0/18:0	t18:1/22:0	t18:0/22:0	d18:1/24:0	d18:0/24:0	t18:1/24:1	t18:1/24:0	t18:0/24:0	d18:0/26:0	t18:1/26:1	t18:1/26:0	t18:0/26:0
Control 0 h	0.43940115	4.52499421	0	0	0.021838792	0.16912341	0.064988582	0	0.090871644	0.08542199	0.020666148	0.009770636	0.109109679	0.538710036	0.366958727	0	0.05930965	0.514552564	0.182548589
Control 1 h	0.17427946	0.9406962	0	0	0	0.147080464	0.033939693	0	0.080927761	0.078790265	0.01276248	0	0.103562838	0.514534743	0.306663416	0	0.056117208	0.537740224	0.159673876
Control 3 h	0.11099436	0.56397683	0	0	0	0.206387602	0.046997562	0	0.119650243	0.120266747	0.018598347	0	0.195485439	0.970882982	0.571184066	0	0.106178372	1.05460774	0.309011366
Control 6 h	0.11313618	0.69967867	0	0	0	0.167898849	0.036621698	0	0.114740279	0.09509821	0.017870834	0	0.150351538	0.722418992	0.407229146	0	0.085138929	0.775084697	0.184451132
Control 24 h	5.20510146	0.94858114	0	0	0.043896812	0.197807632	0.118374599	0	0.102828969	0.158377384	0.020670114	0.013222348	0.111587606	0.611587426	0.643206531	0	0.07428802	0.675407348	0.254695655
Control 48 h	11.5882316	1.44470327	0	0	0.048710247	0.28417894	0.187815991	0	0.127198861	0.153673221	0.035760673	0.016477333	0.172622632	0.74122574	0.914168852	0	0.092154083	0.829998719	0.327969796
nmol/g[FW]																			
0 h	0.43940115	4.52499421	0.060136878	0	0.021838792	0.16912341	0.064988582	0	0.090871644	0.08542199	0.020666148	0.009770636	0.109109679	0.538710036	0.366958727	0	0.05930965	0.514552564	0.182548589
1 h	6.29954943	4.51537375	0.120143967	0	0	0.222909325	0.073464772	0	0.180449265	0.170781037	0.084255258	0.020927777	0.203243387	1.164885276	0.728435242	0	0.146711281	1.293370537	0.342443554
3 h	7.40246282	4.92252897	0.275645516	0	0	0.148498716	0.0498743	0	0.086763379	0.107658606	0.013705855	0.005091845	0.126646705	0.638928653	0.435359106	0	0.078345839	0.588286124	0.203158529
6 h	9.47007204	2.78357739	0.142962715	0	0	0.28682991	0.079879842	0	0.236092042	0.161182828	0.074120765	0.027927832	0.276867905	1.14945	0.771524512	0	0.126966576	1.282310457	0.394231116
24 h	19.2259674	3.24874324	0.331870022	0	0.051976085	0.317243553	0.213962284	0	0.216847206	0.245563227	0.071702405	0.027793326	0.269327492	1.349059317	1.312606511	0	0.176073213	1.385479726	0.578747282
48 h	55.8301618	4.06187852	1.550167114	0	0.068259658	0.276153558	0.229440153	0	0.16619177	0.197430576	0.036347794	0.018921677	0.182838345	0.860583998	1.09034416	0	0.098825654	0.938904229	0.470554991

	D7 sphingobases/ceramides																		
nmol/g[FW]																			
	D7-d18:0	D7-t18:0	D7-d18:0P	D7-t18:0P	D7-d18:0/16:0	D7-t18:1/16:0	D7-t18:0/16:0	D7-d18:0/18:0	D7-t18:1/22:0	D7-t18:0/22:0	D7-d18:1/24:0	D7-d18:0/24:0	D7-t18:1/24:1	D7-t18:1/24:0	D7-t18:0/24:0	D7-d18:0/26:0	D7-t18:1/26:1	D7-t18:1/26:0	D7-t18:0/26:0
0 h	0	0	0	0	0	0	0	0	0	0	0	0	0	0	0	0	0	0	0
1 h	203.273012	1.1160428	0.414829476	0.089095152	0.016941441	0.004886684	0.005584568	0	0	0.007529495	0	0.010518446	0	0	0.012189521	0.002922927	0	0	0.007081329
3 h	250.75448	2.88919167	1.133281569	0.15888423	0.033631576	0.006983949	0.013523284	0.00291908	0.005046997	0.016139028	0	0.01720335	0	0.007337338	0.024848251	0.00491479	0	0.011647043	0.014349477
6 h	251.940595	5.36583913	3.059006522	0.384682102	0.143858443	0.043510689	0.056017735	0.010416865	0.015233999	0.073855102	0	0.097453122	0.007624784	0.037018001	0.092264559	0.023363821	0	0.04079511	0.06244519
24 h	286.586572	2.20890502	3.746685134	0.306009535	0.292301129	0.049121425	0.108411441	0.006853062	0.009571827	0.068075212	0	0.066944356	0.009606345	0.03439721	0.112952074	0.004375276	0	0.036201507	0.043038147
48 h	274.291941	1.92532226	6.619595917	0.43761337	0.260830942	0.034779989	0.080826874	0.013038001	0.007727916	0.040461791	0	0.041274409	0.005763891	0.022279857	0.080746066	0.006523743	0	0.023124433	0.03066542

nmol/g[FW]	D7 sphingobases/ceramides																	
	D7-d18:0	D7-t18:0	D7-d18:0P	D7-t18:0P	D7-d18:0/16:0	D7-t18:1/16:0	D7-t18:0/16:0	D7-d18:0/18:0	D7-t18:1/22:0	D7-t18:0/22:0	D7-d18:1/24:0	D7-d18:0/24:0	D7-t18:1/24:1	D7-t18:1/24:0	D7-t18:0/24:0	D7-d18:0/26:0	D7-t18:1/26:1	D7-t18:1/26:0	D7-t18:0/26:0
0 h	0	0	0	0	0	0	0	0	0	0	0	0	0	0	0	0	0	0	0
1 h	203.273012	1.1160428	0.414829476	0.089095152	0.016941441	0.004886684	0.005584568	0	0	0.007529495	0	0.010518446	0	0	0.012189521	0.002922927	0	0	0.007081329
3 h	250.7544803	2.889191672	1.133281569	0.15888423	0.033631576	0.006983949	0.013523284	0.00291908	0.005046997	0.016139028	0	0.01720335	0	0.007337338	0.024848251	0.00491479	0	0.011647043	0.014349477
6 h	251.9405953	5.365839128	3.059006522	0.384682102	0.143858443	0.043510689	0.056017735	0.010416865	0.015233999	0.073855102	0	0.097453122	0.007624784	0.037018001	0.092264559	0.023363821	0	0.04079511	0.06244519
24 h	286.5865724	2.208905018	3.746685134	0.306009535	0.292301129	0.049121425	0.108411441	0.006853062	0.009571827	0.068075212	0	0.066944356	0.009606345	0.03439721	0.112952074	0.004375276	0	0.036201507	0.043038147
48 h	274.2919408	1.925322259	6.619595917	0.43761337	0.260830942	0.034779989	0.080826874	0.013038001	0.007727916	0.040461791	0	0.041274409	0.005763891	0.022279857	0.080746066	0.006523743	0	0.023124433	0.03066542

nmol/g[FW]	sphingobases				ceramides														
	d18:0	t18:0	d18:0P	t18:0P	d18:0/16:0	t18:1/16:0	t18:0/16:0	d18:0/18:0	t18:1/22:0	t18:0/22:0	d18:1/24:0	d18:0/24:0	t18:1/24:1	t18:1/24:0	t18:0/24:0	d18:0/26:0	t18:1/26:1	t18:1/26:0	t18:0/26:0
0 h	0.439401154	4.524994206	0.060136878	0	0.021838792	0.16912341	0.064988582	0	0.090871644	0.08542199	0.020666148	0.009770636	0.109109679	0.538710036	0.366958727	0	0.05930965	0.514552564	0.182548589
1 h	6.299549434	4.515373751	0.120143967	0	0	0.222909325	0.073464772	0	0.180449265	0.170781037	0.084255258	0.020927777	0.203243387	1.164885276	0.728435242	0	0.146711281	1.293370537	0.342443554
3 h	7.402462816	4.92252897	0.275645516	0	0	0.148498716	0.0498743	0	0.086763379	0.107658606	0.013705855	0.005091845	0.126646705	0.638928653	0.435359106	0	0.078345839	0.588286124	0.203158529
6 h	9.470072036	2.783577386	0.142962715	0	0	0.28682991	0.079879842	0	0.236092042	0.161182828	0.074120765	0.027927832	0.276867905	1.14945	0.771524512	0	0.126966576	1.282310457	0.394231116
24 h	19.22596742	3.248743239	0.331870022	0	0.051976085	0.317243553	0.213962284	0	0.216847206	0.245563227	0.071702405	0.027793326	0.269327492	1.349059317	1.312606511	0	0.176073213	1.385479726	0.578747282
48 h	55.83016183	4.061878522	1.550167114	0	0.068259658	0.276153558	0.229440153	0	0.16619177	0.197430576	0.036347794	0.018921677	0.182838345	0.860583998	1.09034416	0	0.098825654	0.938904229	0.470554991

Control 0 h	0.439401154	4.524994206	0	0	0.021838792	0.16912341	0.064988582	0	0.090871644	0.08542199	0.020666148	0.009770636	0.109109679	0.538710036	0.366958727	0	0.05930965	0.514552564	0.182548589
Control 1 h	0.174279463	0.940696203	0	0	0	0.147080464	0.033939693	0	0.080927761	0.078790265	0.01276248	0	0.103562838	0.514534743	0.306663416	0	0.056117208	0.537740224	0.159673876
Control 3 h	0.110994361	0.563976835	0	0	0	0.206387602	0.046997562	0	0.119650243	0.120266747	0.018598347	0	0.195485439	0.970882982	0.571184066	0	0.106178372	1.05460774	0.309011366
Control 6 h	0.11313618	0.699678668	0	0	0	0.167898849	0.036621698	0	0.114740279	0.09509821	0.017870834	0	0.150351538	0.722418992	0.407229146	0	0.085138929	0.775084697	0.184451132
Control 24 h	5.205101461	0.948581137	0	0	0.043896812	0.197807632	0.118374599	0	0.102828969	0.158377384	0.020670114	0.013222348	0.111587606	0.611587426	0.643206531	0	0.07428802	0.675407348	0.254695655
Control 48 h	11.5882316	1.444703267	0	0	0.048710247	0.28417894	0.187815991	0	0.127198861	0.153673221	0.035760673	0.016477333	0.172622632	0.74122574	0.914168852	0	0.092154083	0.829998719	0.327969796

Sum	d18:0	t18:0	d18:0P	t18:0P	d18:0/16:0	t18:1/16:0	t18:0/16:0	d18:0/18:0	t18:1/22:0	t18:0/22:0	d18:1/24:0	d18:0/24:0	t18:1/24:1	t18:1/24:0	t18:0/24:0	d18:0/26:0	t18:1/26:1	t18:1/26:0	t18:0/26:0
0 h	0.439401154	4.524994206	0.060136878	0	0.021838792	0.16912341	0.064988582	0	0.090871644	0.08542199	0.020666148	0.009770636	0.109109679	0.538710036	0.366958727	0	0.05930965	0.514552564	0.182548589
1 h	209.5725615	5.63141655	0.534973443	0.089095152	0.016941441	0.22779601	0.07904934	0	0.180449265	0.178310532	0.084255258	0.031446222	0.203243387	1.164885276	0.740624762	0.002922927	0.146711281	1.293370537	0.349524882
3 h	258.1569431	7.811720641	1.408927085	0.15888423	0.033631576	0.155482665	0.063397584	0.00291908	0.091810376	0.123797634	0.013705855	0.022295196	0.126646705	0.646265991	0.460207357	0.00491479	0.078345839	0.599933167	0.217508006
6 h	261.4106673	8.149416514	3.201969237	0.384682102	0.143858443	0.3303406	0.135897577	0.010416865	0.251326042	0.23503793	0.074120765	0.125380954	0.284492688	1.186468	0.863789071	0.023363821	0.126966576	1.323105567	0.456676306
24 h	305.8125398	5.457648257	4.078555156	0.306009535	0.344277214	0.366364978	0.322373725	0.006853062	0.226419033	0.313638439	0.071702405	0.094737683	0.278933837	1.383456527	1.425558585	0.004375276	0.176073213	1.421681233	0.621785429
48 h	330.1221026	5.987200782	8.169763032	0.43761337	0.3290906	0.310933547	0.310267028	0.013038001	0.173919686	0.237892368	0.036347794	0.060196087	0.188602236	0.882863855	1.171090226	0.006523743	0.098825654	0.962028662	0.501220411

% deut																			
0 h	0	0	0	0	0	0	0	0	0	0	0	0	0	0	0	0	0	0	0
1 h	96.99409627	19.81815392	77.54206898	100	100	2.145201939	7.064661248	0	0	4.222686593	0	33.44899592	0	0	1.645842978	100	0	0	2.025986998
3 h	97.13257264	36.98534298	80.4357856	100	100	4.491786158	21.3309145	100	5.497197214	13.03662043	0	77.16169249	0	1.135343383	5.399359722	100	0	1.941390061	6.597217732
6 h	96.37731997	65.84323085	95.53516276	100	100	13.17146281	41.2205548	100	6.061448851	31.42263138	0	77.72561869	2.680133406	3.120016768	10.68137605	100	0	3.083284607	13.67384067
24 h	93.7131527	40.47356873	91.8630493	100	84.90283918	13.40778395	33.62911811	100	4.22748348	21.7049964	0	70.66286025	3.44395097	2.486323849	7.923355446	100	0	2.546387062	6.921704023
48 h	83.08802671	32.15730238	81.02555596	100	79.2580955	11.1856663	26.05074571	100	4.443381714	17.0084444	0	68.566599	3.056109772	2.52358926	6.894948331	100	0	2.403715598	6.118150704

100

Basal level																			
0 h	100	100	100	0	100	100	100	0	100	100	100	100	100	100	100	0	100	100	100
1 h	3.005903726	80.18184608	22.45793102	0	0	97.85479806	92.93533875	0	100	95.77731341	100	66.55100408	100	100	98.35415702	0	100	100	97.974013
3 h	2.867427359	63.01465702	19.5642144	0	0	95.50821384	78.6690855	0	94.50280279	86.96337957	100	22.83830751	100	98.86465662	94.60064028	0	100	98.05860994	93.40278227
6 h	3.622680028	34.15676915	4.46483724	0	0	86.82853719	58.7794452	0	93.93855115	68.57736862	100	22.27438131	97.31986659	96.87998323	89.31862395	0	100	96.91671539	86.32615933
24 h	6.286847305	59.52643127	8.136950696	0	15.09716082	86.59221605	66.37088189	0	95.77251652	78.2950036	100	29.33713975	96.55604903	97.51367615	92.07664455	0	100	97.45361294	93.07829598
48 h	16.91197329	67.84269762	18.97444404	0	20.7419045	88.8143337	73.94925429	0	95.55661829	82.9915556	100	31.433401	96.94389023	97.47641074	93.10505167	0	100	97.5962844	93.8818493

For three non-labelled compounds, no metabolites could be detected. For a conservative calculation of % labelling, we used the respective limits of detection (LOD):

nmol/g[FW]	Sphingobase	Ceramides	
	t18:0P	d18:0/18:0	d18:0/26:0
0 h	0.038	0.009	0.008
1 h	0.038	0.009	0.008
3 h	0.038	0.009	0.008
6 h	0.038	0.009	0.008
24 h	0.038	0.009	0.008
48 h	0.038	0.009	0.008
