# Supplementary material for: Sphingolipid Long-Chain Base Phosphate Degradation Can Be a Rate-Limiting Step in Long-Chain Base Homeostasis
Source: Front Plant Sci. 2022 Jun 15;13:911073. doi: 10.3389/fpls.2022.911073 (PMC9240600; doi:10.3389/fpls.2022.911073)
Supplement: Supplementary File 1 — Detailed description of sphingolipid detection and quantification by LCMS, including supplementary references. [file Table_2.pdf]

Abbreviation	Common Name	Systematic Name	Species Shorthand	Lipid Maps ID	Source (Company)	product # (company)	Purity
d18:0	Sphinganine	Sphinganine	SPB 18:0;O2	LMSP01020001	Avanti Polar Lipids	860498P	>99%
d18:1	Sphingosine	Sphing-4-enine	SPB 18:1;O2	LMSP01010001	Avanti Polar Lipids	860490P	>99%
t18:0	Phytosphingosine	4R-hydroxysphinganine	SPB 18:0;O3	LMSP01030001	Avanti Polar Lipids	860499P	>99%
d20:1	N,N-dimethylsphingosine	N,N-dimethylsphing-4-enine	SPB 20:1;O2	LMSP01070001	Avanti Polar Lipids	860660P	>99%
d18:1P	Sphingosine-1-phosphate	Sphing-4-enine-1-phosphate	SPBP 18:1;O2	LMSP01050001	Avanti Polar Lipids	860492P	>99%
d18:0P	Sphinganine-phosphate	Sphinganine-1-phosphate	SPBP 18:0;O2	LMSP01050002	Avanti Polar Lipids	860536P	>99%
t18:0P	Phytosphingosine 1-phosphate	(2S,3S,4R)-2-amino-3,4-dihydroxyoctadecyl dihydrogen phosphate	SPBP 18:0;O3	LMSP01050003	Avanti Polar Lipids	860491P	>99%
d17:1	C17 Sphingosine	Heptadeca-sphing-4-enine	SPB 17:1;O2	LMSP01040002	Avanti Polar Lipids	860640P	>99%
d17:1P	C17 Sphingosine-1-phosphate	heptadeca-sphing-4-enine-1-phosphate	SPBP 17:1;O2	LMSP01050007	Avanti Polar Lipids	860641P	>99%
D_7-d18:0	Sphinganine-d7	D-*erythro*-Sphinganine-d7			Avanti Polar Lipids	860658P	>99%
Cer(d18:0/16:0)	Cer(d18:0/16:0)	N-(hexadecanoyl)-sphinganine	Cer 34:0;O2	LMSP02020001	Avanti Polar Lipids	860634P	>99%
Cer(d18:0/18:0)	Cer(d18:0/18:0)	N-(octadecanoyl)-sphinganine	Cer 36:0;O2	LMSP02020008	Avanti Polar Lipids	860627P	>99%
Cer(d18:0/18:1)	Cer(d18:0/18:1(9Z))	N-(9Z-octadecenoyl)-sphinganine	Cer 36:1;O2	LMSP02020015	Avanti Polar Lipids	860624P	>99%
Cer(d18:0/24:0)	Cer(d18:0/24:0)	N-(tetracosanoyl)-sphinganine	Cer 42:0;O2	LMSP02020012			
Cer(d18:0/24:1)	Cer(d18:0/24:1(15Z))	N-(15Z-tetracosenoyl)-sphinganine	Cer 42:1;O2	LMSP02020011	Avanti Polar Lipids	860629P	>99%
Cer(d18:0/26:0)	Cer(d18:0/26:0)	N-(hexacosanoyl)-sphinganine	Cer 44:0;O2	LMSP02020014			
Cer(d18:1/10:0)	Cer(d18:1/10:0)	N-decanoyl-D-*erythro*-sphingosine	Cer 28:1;O2		Avanti Polar Lipids	860510P	>99%
Cer(d18:1/24:0)	Cer(d18:1/24:0)	N-(tetracosanoyl)-sphing-4-enine	Cer 42:1;O2	LMSP02010012	Avanti Polar Lipids	860524P	>99%

Cer(t18:0/16:0)	Cer(t18:0/16:0)	N-(hexadecanoyl)-4R-hydroxysphinganine	Cer 34:0;O3	LMSP02030001	Avanti Polar Lipids	860617P	>99%
Cer(t18:0/22:0)	Cer(t18:0/22:0)	N-(docosanoyl)-4R-hydroxysphinganine	Cer 40:0;O3	LMSP02030008			
Cer(t18:0/24:0)	Cer(t18:0/24:0)	N-(tetracosanoyl)-4R-hydroxysphinganine	Cer 42:0;O3	LMSP02030004	Avanti Polar Lipids	860724P	>99%
Cer(t18:0/26:0)	Cer(t18:0/26:0)	N-(hexacosanoyl)-4R-hydroxysphinganine	Cer 44:0;O3	LMSP02030005			
Cer(t18:1/16:0)	Cer(t18:1/16:0)		Cer 34:1;O3				
Cer(t18:1/22:0)	Cer(t18:1/22:0)		Cer 40:1;O3				
Cer(t18:1/24:0)	Cer(t18:1/24:0)		Cer 42:1;O3				
Cer(t18:1/24:1)	Cer(t18:1/24:1)		Cer 42:2;O3				
Cer(t18:1/26:0)	Cer(t18:1/26:0)		Cer 44:1;O3				
Cer(t18:1/26:1)	Cer(t18:1/26:1)		Cer 44:2;O3				
	Acetonitrile	Acetonitrile			Biosolve	0001207802BS	LC-MS grade
	Ammonium acetate	Ammonium acetate			Biosolve	0001244156BS	UPLC/MS-CC/SFC grade
	Ammonium formate	Ammonium formate			VWR chemicals	21254.260	$\geq$97%
	1-Butanol	Butan-1-ol			VWR chemicals	20810.323	99.8%
	Citric acid monohydrate	2-Hydroxypropan-1,2,3-tricarbonsäure			Merck	1.00242.5000	
DMSO	Dimethylsulfoxide	Dimethylsulfoxide			Merck	1029310500	H_2O content $\leq$ 0.025%
FB_1	Fumonisin B1	Fumonisin B1		LMSP01080022	Sigma-Aldrich/ Merck	F1147	$\geq$98%
	Formic acid	Formic acid			Biosolve	0006914143BS	UPLC/MS-CC/SFC
MeOH	Methanol	Methyl alcohol			Merck	1.06007.2500	LiChrosolv gradient grade for LC
	Isopropanol	Propan-2-ol			Biosolve	0016264102BS	LC-MS grade
NaH_2PO_4	Sodium dihydrogen phosphate -monohydrate				Merck	1.06346.1000	p.a.

D-*erythro*-Sphinganine-d_7

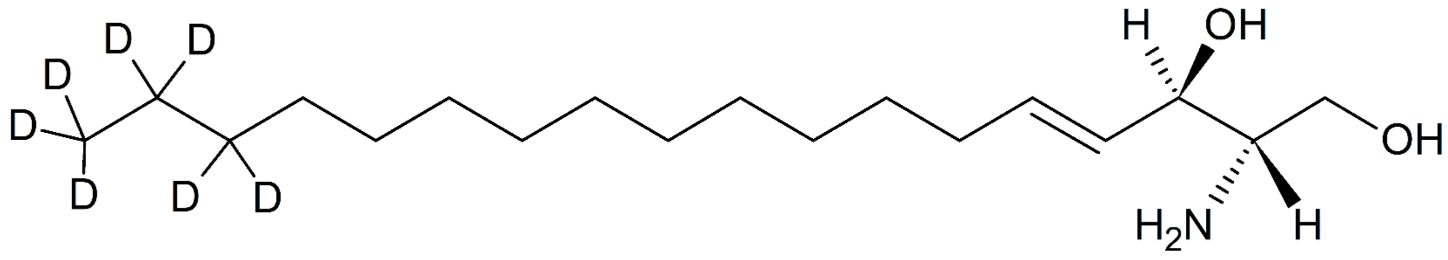

Lipid Maps reference:

Fahy E., Sud M., Cotter D. & Subramaniam S. (2007) LIPID MAPS® online tools for lipid research. Nucleic Acids Research 35: W606-12.

Sud M., Fahy E., Cotter D., Brown A., Dennis E.A., Glass C.K., Merrill A.H. Jr, Murphy R.C., Raetz C.R., Russell D.W., Subramaniam S. (2007) LMSD: LIPID MAPS® structure database. Nucleic Acids Research 35: D527-32.
